# High-risk HPV type-specific clearance rates in cervical screening

**DOI:** 10.1038/sj.bjc.6603653

**Published:** 2007-03-06

**Authors:** N W J Bulkmans, J Berkhof, S Bulk, M C G Bleeker, F J van Kemenade, L Rozendaal, P J F Snijders, C J L M Meijer

**Affiliations:** 1 Department of Pathology, VU University Medical Center, Amsterdam, The Netherlands; 2 Department of Clinical Epidemiology and Biostatistics, VU University Medical Center, Amsterdam, The Netherlands

**Keywords:** human papillomavirus, clearance, type, cervical screening, cervical intraepithelial neoplasia

## Abstract

We assessed clearance rates of 14 high-risk human papillomavirus (hrHPV) types in hrHPV-positive women with normal cytology and borderline/mild dyskaryosis (BMD) in a population-based cervical screening cohort of 44 102 women. The 6-month hrHPV type-specific clearance rates, that is, clearance of the same type as detected at baseline, in women with normal and BMD smears were 43% (95% confidence interval (CI) 39–47) and 29% (95% CI 24–34), respectively. Corresponding 18-month clearance rates were markedly higher, namely 65% (95% CI 60–69) and 41% (95% CI 36–47), respectively. The lowest clearance rates in women with normal cytology were observed for HPV16, HPV18, HPV31, and HPV33. Significantly reduced 18-month clearance rates at a significance level of 1% were observed for HPV16 (49%, 95% CI 41–59) and HPV31 (50%, 95% CI 39–63) in women with normal cytology, and for HPV16 (19%, 95% CI 12–29) in women with BMD. Among women who did not clear hrHPV, women with HPV16 persistence displayed an increased detection rate of ⩾CIN3 (normal *P*<0.0001; BMD, *P*=0.005). The type-specific differences in clearance rates indicate the potential value of hrHPV genotyping in screening programs. Our data support close surveillance (i.e. referral directly, or within 6 months) of women with HPV16 and are inconclusive for surveillance of women with HPV18, HPV31, and HPV33. For the other hrHPV-positive women, it seems advisable to adopt a conservative management with a long waiting period, as hrHPV clearance is markedly higher after 18 months than after 6 months and the risk for ⩾CIN3 is low.

Persistent infection with high-risk human papillomavirus (hrHPV) is the primary cause for the development of cervical carcinoma (Walboomers *et al*, 1999; Bosch *et al*, 2002). Several studies have shown that hrHPV testing can improve identification of women who have or will develop high-grade cervical intraepithelial neoplasia or cervical cancer (⩾CIN2) (Clavel *et al*, 2001; Kulasingam *et al*, 2002; Cuzick *et al*, 2003; Peto *et al*, 2004; Kotaniemi-Talonen *et al*, 2005; Cuzick *et al*, 2006).

In the search of an optimal screening algorithm using hrHPV testing, it is essential to determine the time point at which the majority of the screening participants has cleared the virus and can be referred back to regular screening. Also, future screening may involve genotyping as different hrHPV types show markedly different risks of high-grade CIN. Particularly HPV16-positive women are more likely to develop ⩾CIN2 than hrHPV-positive women infected with a non-HPV16 type (Bulkmans *et al*, 2005; Castle *et al*, 2005; Khan *et al*, 2005; Berkhof *et al*, 2006). So far, little is known about type-specific clearance rates of hrHPV infections. Some studies reported relatively low clearance of HPV16 infections compared with other high-risk HPV infections, but samples sizes were small and results were not statistically significant (Ho *et al*, 1998; Molano *et al*, 2003; Richardson *et al*, 2003).

To obtain information about the course of 14 different hrHPV types, we investigated repeated hrHPV typing results collected from a large population-based screening cohort. We assessed differences among the 14 hrHPV types in clearance and in the occurrence of ⩾CIN3.

## MATERIALS AND METHODS

### Study population

From January 1999 to September 2002, 44 102 women between 30 and 60 years of age invited for the regular Dutch cervical screening programme participated in the Population-Based Screening Amsterdam (POBASCAM) trial (Bulkmans *et al*, 2004). In this prospective randomised controlled trial, the efficacy of hrHPV testing in conjunction with cytology (intervention group) is compared with that of classical cytology alone (control group, hrHPV results blinded) in the setting of population-based cervical screening. The design of the POBASCAM trial and the baseline results have been described previously (Bulkmans *et al*, 2004). All women gave informed consent and the study was approved by both the Medical Ethics Committee of the VU University Medical Center (no. 96/103) and the Ministry of Public Health (VWS. no 328650). The study has been registered at the International Trial Register (ISRCTN20781131).

We included all hrHPV-positive participants who were advised to return for repeat testing at 6 and 18 months according to the study design, that is, participants with normal cytology from the intervention group (*n*=763) and participants with borderline or mild dyskaryosis (BMD) from the intervention (*n*=185) and control (*n*=196) group. Borderline/mild dyskaryosis is equivalent to ASC-US, ASC-H, LSIL, AGC and AGC favour neoplastic, according to the Bethesda 2001 classification (Bulk *et al*, 2004). Women who were positive for hrHPV by generic hrHPV test, but negative after typing, were excluded from the analysis (*n*=57), leaving 713 hrHPV-positive participants with normal cytology carrying 865 hrHPV infections, and 374 hrHPV-positive participants with BMD carrying 491 hrHPV infections.

**Figure 1:**
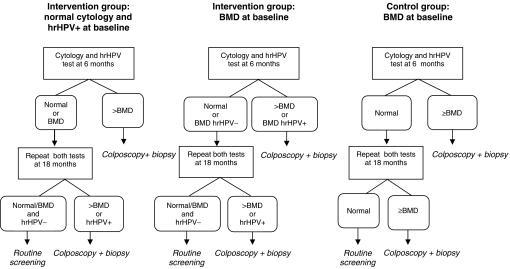
Management of women in the POBASCAM study who were advised to return for repeat testing at 6 and 18 months.

For all included participants, hrHPV testing and typing were performed both at baseline and at 6 and 18 months of follow-up. Participants with a baseline normal smear were referred for colposcopy at 6 months in case the repeat smear displayed moderate dyskaryosis or worse (>BMD; equivalent to HSIL according to Bethesda 2001; Bulk *et al*, 2004). Participants with baseline BMD were referred for colposcopy at 6 months in case the repeat smear result was ⩾BMD in the control group and either >BMD or hrHPV-positive BMD in the intervention group. A flowchart of the screening management of women who were advised to return for repeat testing is presented in Figure 1.

### Cytology and hrHPV testing

Conventional cytological smears were prepared with a Cervex brush® and classified according to the Dutch CISOE-A classification, which can be translated to the Bethesda 2001 classification (Bulk *et al*, 2004). After taking the smear, the brush was placed in a vial containing collection medium (i.e. 5 ml PBS and 0.5% thiomersal) for hrHPV-DNA testing. Detection of hrHPV-DNA was performed by a generic hrHPV test, that is, GP5+/6+ PCR-enzyme immunoassay (GP5+/6+ PCR EIA), using a cocktail of 14 high-risk types, that is, 16, 18, 31, 33, 35, 39, 45, 51, 52, 56, 58, 59, 66, and 68 (Jacobs *et al*, 1997). GP5+/6+ PCR EIA-positive cases were subsequently typed by reverse line blotting (RLB) (van den Brule *et al*, 2002). Interpretation of cytology and hrHPV testing by technicians was performed blinded to the other test result.

### Statistical analysis

We calculated the clearance rates of 14 hrHPV types with type-specific clearance defined as a negative RLB test result for that HPV type in the follow-up smear. Women treated for ⩾CIN2 were considered not to have cleared the virus during the study period of 18 months. Participants were censored if lost to follow-up, or if a biopsy was taken with CIN0/1 as histological outcome. Time was set equal to the target repeat date (i.e. 6 or 18 months). The 18-month clearance rates were estimated by Kaplan–Meier method. The clearance rates were accompanied by 95% confidence intervals (95% CI) (Klein and Moeschberger, 1997). Data were stratified in three age categories corresponding to the age at the first round, second round, and at rounds 3–7 in nationwide screening (i.e. 29–33, 34–38, and 39–60 years). Differences in 6-month clearance rates were assessed by Cochran's Mantel–Haenszel test and differences in 18-month clearance rates were assessed by stratified log-rank testing (Heimann and Neuhaus, 1998). The level of statistical significance was set at 0.01. The main analyses did not distinguish whether the hrHPV infection was observed in a women with single or multiple hrHPV types. Analyses were repeated for women with single hrHPV infections only. To examine an effect of coexisting hrHPV infections on clearance, type-specific clearance rates in single and multiple infections were compared by stratified log-rank testing. For clinical practice it might be more feasible to define clearance as a negative hrHPV follow-up test result for any hrHPV type. Therefore, analyses were repeated with clearance defined as a negative generic hrHPV test result.

To assess the hrHPV type-specific risk for ⩾CIN3 in women who did not clear the hrHPV infection (ie. viral persistence), participants were selected that revealed RLB positivity during follow-up for at least one hrHPV type detected at baseline. The association between persistent hrHPV type and ⩾CIN3 was assessed by Cochran's Mantel–Haenszel test stratified for age. To examine the effect of coexisting hrHPV infections, ⩾CIN3 rates in women with single and multiple hrHPV infections were compared by Cochran's Mantel–Haenszel testing, stratified for age, cytology, and HPV-type. All analyses were performed using SPSS12.0.

## RESULTS

### Study subjects

The mean ages of hrHPV-positive women with normal cytology and BMD at baseline were 38.3 years (range 29–60 years) and 36.2 years (range 29–59 years), respectively. Of women with normal cytology, 23.1% (165/713) did not respond to the follow-up invitation at 6 months and 28.0% (146/522) did not respond to the second follow-up invitation at 18 months. For women with BMD, the nonresponse rates to follow-up invitations at 6 and 18 months were 9.9% (37/374) and 28.8% (53/184), respectively. Loss to follow-up was not hrHPV type specific (*P*>0.05 for each type). Multiple hrHPV infections were less prevalent in women with normal cytology than in women with BMD (18.0 *vs* 24.6%, *P*=0.011).

### Type-specific clearance rates for hrHPV infections in normal cytology

The 6- and 18-month clearance rates of the different hrHPV types in women with normal cytology at baseline are presented in Table 1. The overall type-specific hrHPV clearance rates at 6 and 18 months were 43% (95% CI 39–47) and 65% (95% CI 60–69).

HPV16 infections displayed a significantly lower 18-month clearance rate (49%, 95% CI 41–59) than other hrHPV infections (69%, 95% CI 65–74; *P*=0.002). The second, third, and fourth lowest 18-month clearance rates were observed for HPV31, HPV33, and HPV18 infections, respectively, but the clearance rate was significantly reduced only for HPV31 (*P*=0.008). When comparing single and multiple infections at baseline, none of the hrHPV types showed marked differences in clearance rates (data not shown). Notably, 18-month clearance for HPV16 was 48% (95% CI 38–58) in women with a single infection and 56% (95% CI 39–75) in women with a multiple infection (*P*=0.310).

When defining clearance as a negative generic hrHPV test result instead of a negative RLB result for a specific hrHPV type, overall hrHPV clearance rates at 6 and 18 months were slightly lower, that is, 36% (95% CI 31–41) and 56% (95% CI 52–60), respectively. The lowest clearance rates were again found for HPV16, HPV18, HPV31, and HPV33 infections.

### Type-specific clearance rates for hrHPV infections in BMD

For hrHPV infections in women with a BMD smear at baseline, 6 and 18-month type-specific clearance rates were 29% (95% CI 24–34) and 41% (95% CI 36–47) (Table 2).

As was the case for women with normal cytology, HPV16 infections in women with BMD showed a significantly reduced 18-month clearance rate (19%, 95% CI 12–29) compared with other hrHPV infections (49%, 95% CI 43–56; *P*<0.0001). For HPV31 and HPV33 infections in BMD, clearance rates were also low, but rates were not significantly lower than for other hrHPV infections. Similar clearance rates were observed when the analysis was repeated for women with single infections only. None of the hrHPV types showed marked differences in clearance rates when comparing single infections at baseline to multiple infections at baseline (data not shown).

When clearance was defined as a negative generic hrHPV test instead of a negative RLB test for a specific hrHPV type, hrHPV clearance rates at 6 and 18 months were 25% (95% CI 19–31) and 32% (95% CI 26–38), respectively. The three types with the lowest 18-month clearance rates were again HPV16, HPV31, and HPV33.

### High-grade lesions in women with persistent hrHPV infections

The 18-month hrHPV type-specific detection rate of ⩾CIN3 in women who showed persistence for at least one hrHPV type was 12% (95% CI 9–17) when baseline cytology was read as normal and 26% (95% CI 19–33) in case of BMD (Table 3). Women with a persistent HPV16 infection had significantly increased 18-month ⩾CIN3 detection rates (25% for baseline normal cytology (95% CI 17–36; *P*<0.0001) and 38% for BMD (95% CI 27–51; *P*=0.005), respectively). Similar results were obtained when repeating the analysis only for women with a single infection at baseline. After exclusion of women with HPV16, marginal increases in ⩾CIN3 detection rates (*P*<0.1) were found in women with HPV18 and HPV31 persistence. It should be noted, however, that subgroups were small.

Interestingly, for normal cytology and BMD combined, the ⩾CIN3 detection rate in women with persistence of at least one hrHPV type was higher in women with a single hrHPV infection (20%, 95% CI 15–25) than in women with multiple hrHPV infections (12%, 95% CI 7–20; *P*=0.015). This difference was most pronounced in women with HPV16 at baseline, as the ⩾CIN3 rate was 35% (95% CI 26–44) when carrying a single infection at baseline and 14% (95% CI 6–32) when carrying multiple infections at baseline (*P*=0.022).

### DISCUSSION

In our population-based screening cohort, we studied the type-specific clearance in women with an hrHPV infection. Overall 18-month clearance rates in women with normal cytology at baseline were about 1.5 times higher than those in women with BMD (65 *vs* 41%). Besides, about one-third of the women who cleared the virus within 18 months showed clearance between 6 and 18 months. The lowest clearance rates were observed for HPV16, HPV18, HPV31, and HPV33. Only HPV16 and HPV31 were statistically distinct from the other hrHPV types in women with normal cytology, and only HPV16 and HPV33 were statistically distinct in women with BMD. The relatively low clearance rate of HPV16 could also have been deduced from earlier studies (Ho *et al*, 1998; Molano *et al*, 2003; Richardson *et al*, 2003). However, in these studies, sample sizes were generally smaller, and either results did not reach statistical significance or many HPV types were grouped together. Furthermore, we found that among women who did not clear the HPV infection, HPV16-positive women displayed ⩾CIN3 lesions more often than women who had a persistent infection with another hrHPV type. Finally, we found that the ⩾CIN3 detection rate in women with viral persistence was higher in women with a single hrHPV infection than in women with multiple hrHPV infections at baseline. This finding is in accordance with data of other studies, which showed that multiple hrHPV infections decrease in prevalence when comparing hrHPV-positive normal cervices to increasing grades of cervical premalignant disease (Lungu *et al*, 1992; Sasagawa *et al*, 2001; An *et al*, 2003).

Previously described data showed that besides HPV16, HPV18, HPV31, and HPV33 also conferred an increased risk of ⩾CIN3 (Castle *et al*, 2005; Khan *et al*, 2005; Berkhof *et al*, 2006). This likely reflects the combined effects of differences in persistence and oncogenic potential of these types compared with other types and finds support by our data. Whereas HPV16 displayed markedly decreased clearance rates and increased ⩾CIN3 rates in case of persistence in both women with normal cytology and BMD, HPV18, HPV31, and HPV33 showed some, but less pronounced, effect on one or both of these parameters. The effect was either limited to women with normal cytology or to women with BMD, or, in case of ⩾CIN3 rate, only evident after excluding the women having HPV16 infections. The relatively small size of the subgroups of women with type-specific persistence of non-HPV16 types is a likely reason that effects of these types on ⩾CIN3 rate were only marginal.

Distinguishing hrHPV types with a decreased clearance rate may have implications for future screening algorithms. When considering implementation of hrHPV genotyping in cervical screening, it is important to evaluate the time point at which genotyping is performed. Genotyping may well be cost-effective when it is limited to baseline samples, and generic hrHPV testing is applied during follow-up. We also calculated clearance rates when clearance was defined as a negative generic hrHPV test instead of a negative RLB result, and it appeared that clearance rates were only 5–10% lower when assessed by a generic hrHPV test. Besides, reduced clearance rates were found for the same hrHPV types as found by a negative RLB result, that is, HPV16, HPV18, HPV31, and HPV33. Therefore, we feel that information about type-specific clearance in cervical screening can be provided with sufficient accuracy by generic hrHPV testing during follow-up.

For screening algorithms, it is also important to determine the optimal testing moment during follow-up. As about one-third of women with cleared infections revealed clearance between 6 and 18 months, a conservative management with a longer waiting period may be considered, particularly for women with normal cytology, as this is likely to result in less medical procedures. However, the follow-up time point should not only be targeted on the hrHPV clearance time but also on the risk of ⩾CIN3 for the different HPV types (Berkhof *et al*, 2006). Notably, women with either BMD or an HPV16-positive normal smear have a clearly increased risk of ⩾CIN3, and should be recalled early or perhaps even referred for colposcopy at baseline. Owing to the small size of the subgroups, our data are inconclusive concerning the surveillance of women with normal cytology and an HPV18, HPV31, or HPV33 infection. For hrHPV-positive women with normal cytology and without HPV16, HPV18, HPV31, and HPV33, a conservative management with repeat testing at a later time point seems feasible. Currently, our data are incorporated in cost-effectiveness studies to assess the optimal algorithm for the follow-up of hrHPV-positive women with normal cytology or BMD.

## Figures and Tables

**Table 1:**
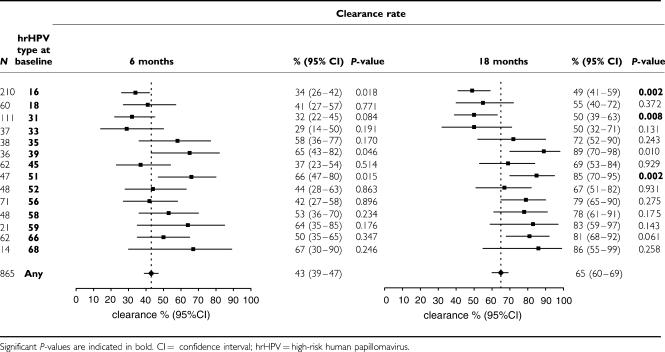
hrHPV-type specific 6- and 18-month clearance rates for hrHPV infections in normal smears

**Table 2:**
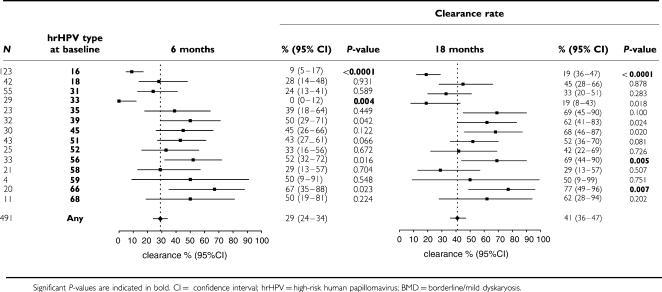
hrHPV-type specific 6- and 18-month clearance rates for hrHPV infections in BMD smears

**Table 3:** HPV type-specific detection rate of ⩾CIN3 lesions in women with normal cytology and with BMD who did not clear the respective HPV type

	**Normal cytology**				**BMD**			
	**Persistent**	**⩾CIN3**			**Persistent**	**⩾CIN3**		
**hrHPV type at baseline**	***N***	**% (95% CI)**	***P*-value**	***P* excl. 16**	***N***	**% (95% CI)**	***P*-value**	***P* excl. 16**
*All hrHPV infections*								
Any	217	12 (9–17)			148	26 (19–33)		
16	76	25 (17–36)	**<0.0001**		60	38 (27–51)	**0.005**	
18	21	19 (8–40)	0.512	*0.061*	13	23 (8–50)	0.820	0.438
31	40	13 (5–26	0.956	*0.169*	16	31 (14–56)	0.550	0.072
33	14	21 (8–48)	0.335	*0.147*	11	9 (2–38)	0.205	0.627
35	5	0 (0–52)	1.000	*1.000*	7	14 (3–51)	0.499	0.969
39	5	0 (0–52)	1.000	*1.000*	7	29 (8–64	0.872	0.396
45	18	0 (0–19)	0.138	*0.596*	11	18 (5–48)	0.573	0.539
51	6	0 (0–46)	1.000	*1.000*	14	21 (8–48)	0.750	0.585
52	14	7 (1–31)	0.541	*1.000*	8	13 (2–47)	0.375	0.860
56	13	0 (0–25)	0.378	*1.000*	10	10 (2–40)	0.255	0.544
58	12	0 (0–26)	0.370	*1.000*	12	8 (1–35)	0.185	0.199
59	3	0 (0–71)	1.000	*1.000*	1	100 (3–100)	1.000	1.000
66	11	0 (0–28)	0.367	*1.000*	4	25 (5–70)	0.999	0.678
68	2	0 (0–84)	1.000	*1.000*	3	0 (0–71)	1.000	1.000
								
*Single hrHPV infections*								
Any	173	13 (9–19)			107	30 (22–39)		
16	59	27 (17–40)	<0.0001		48	44 (31–58)	0.009	
18	14	14 (4–40)	0.924	0.195	5	40 (12–77)	0.660	0.235
31	28	11 (4–27)	0.603	0.285	9	44 (19–73)	0.198	0.039
33	10	20 (6–51)	0.519	0.911	6	17 (3–56)	0.453	0.904
35	3	0 (0–71)	1.000	1.000	4	25 (5–70)	1.00	0.707
39	4	0 (0–60)	1.000	1.000	3	33 (6–79)	0.910	0.476
45	16	0 (0–21)	0.134	0.591	5	20 (4–62)	0.696	0.914
51	4	0 (0–60)	1.000	1.000	7	0 (0–41)	0.100	0.328
52	6	0 (0–46)	1.000	1.000	5	20 (4–62)	0.549	0.929
56	8	0 (0–37)	0.599	1.000	4	0 (0–60)	0.315	1.000
58	11	0 (0–28)	0.363	1.000	9	0 (0–34)	0.055	0.186
59	2	0 (0–84)	1.000	1.000	0	—	—	—
66	8	0 (0–37)	0.599	1.000	0	—	—	—
68	0	—	—	—	2	0 (0–84)	1.000	1.000

Persistent=not cleared in follow-up for the at least one specific hrHPV type.

All hrHPV infections=single and multiple hrHPV infections combined.

Any=positive for any of the 14 hrHPV types.

p excl. 16=*P*-value by Cochran's Mantel–Haenszel test stratified for age, after excluding women persistent for HPV16.

Significant *P*-values are indicated in bold and marginal effects are in italics.

BMD, borderline/mild dyskaryosis; CI, confidence interval; ⩾CIN3, cervical intraepithelial neoplasia grade 3 or worse; hrHPV, high-risk human papillomavirus.

## Appendix

### Population-Based Screening Amsterdam study group collaborators other than authors

K van Groningen (Department of Pathology, Spaarne Ziekenhuis, Hoofddorp), W Ruitinga (Department of Pathology, Kennemer Gasthuis, Haarlem), ME Boon (Leiden Cytology and Pathology Laboratory, Leiden), M van Ballegooijen (Department of Public Health and Social Medicine, Erasmus University Rotterdam), AJP Boeke (Institute for Research in Extramural Medicine, VU University Medical Center, Amsterdam), FJ Voorhorst (Department of Clinical Epidemiology and Biostatistics, VU University Medical Center, Amsterdam), and RHM Verheijen (Department of Obstetrics and Gynaecology, VU University Medical Center, Amsterdam).

## References

[bib1] An HJ, Cho NH, Lee SY, Kim IH, Lee C, Kim SJ, Mun MS, Kim SH, Jeong JK (2003) Correlation of cervical carcinoma and precancerous lesions with human papillomavirus (HPV) genotypes detected with the HPV DNA chip microarray method. Cancer 97: 1672–1680 12655524 10.1002/cncr.11235

[bib2] Berkhof J, Bulkmans NW, Bleeker MC, Bulk S, Snijders PJ, Voorhorst FJ, Meijer CJ (2006) Human papillomavirus type-specific 18-month risk of high-grade cervical intraepithelial neoplasia in women with a normal or borderline/mildly dyskaryotic smear. Cancer Epidemiol Biomarkers Prev 15: 1268–1273 16835322 10.1158/1055-9965.EPI-05-0764

[bib3] Bosch FX, Lorincz A, Munoz N, Meijer CJ, Shah KV (2002) The causal relation between human papillomavirus and cervical cancer. J Clin Pathol 55: 244–265 11919208 10.1136/jcp.55.4.244 PMC1769629

[bib4] Bulk S, Van Kemenade FJ, Rozendaal L, Meijer CJ (2004) The Dutch CISOE-A framework for cytology reporting increases efficacy of screening upon standardisation since 1996. J Clin Pathol 57: 388–393 15047743 10.1136/jcp.2003.011841 PMC1770272

[bib5] Bulkmans NW, Bleeker MC, Berkhof J, Voorhorst FJ, Snijders PJ, Meijer CJ (2005) Prevalence of types 16 and 33 is increased in high-risk human papillomavirus positive women with cervical intraepithelial neoplasia grade 2 or worse. Int J Cancer 117: 177–181 15900579 10.1002/ijc.21210

[bib6] Bulkmans NW, Rozendaal L, Voorhorst FJ, Boeke AJ, Snijders PJ, Meijer CJ (2004) POBASCAM, a population-based randomised controlled trial for implementation of high-risk HPV testing in cervical screening. Int J Cancer 110: 94–101 15054873 10.1002/ijc.20076

[bib7] Castle PE, Solomon D, Schiffman M, Wheeler CM (2005) Human papillomavirus type 16 infections and 2-year absolute risk of cervical precancer in women with equivocal or mild cytologic abnormalities. J Natl Cancer Inst 97: 1066–1071 16030304 10.1093/jnci/dji186

[bib8] Clavel C, Masure M, Bory JP, Putaud I, Mangeonjean C, Lorenzato M, Nazeyrollas P, Gabriel R, Quereux C, Birembaut P (2001) Human papillomavirus testing in primary screening for the detection of high-grade cervical lesions: a study of 7932 women. Br J Cancer 84: 1616–1623 11401314 10.1054/bjoc.2001.1845 PMC2363679

[bib9] Cuzick J, Clavel C, Petry KU, Meijer CJ, Hoyer H, Ratnam S, Szarewski A, Birembaut P, Kulasingam S, Sasieni P, Iftner T (2006) Overview of the European and North American studies on HPV testing in primary cervical cancer screening. Int J Cancer 119: 1095–1101 16586444 10.1002/ijc.21955

[bib10] Cuzick J, Szarewski A, Cubie H, Hulman G, Kitchener H, Luesley D, McGoogan E, Menon U, Terry G, Edwards R, Brooks C, Desai M, Gie C, Ho L, Jacobs I, Pickles C, Sasieni P (2003) Management of women who test positive for high-risk types of human papillomavirus: the HART study. Lancet 362: 1871–1876 14667741 10.1016/S0140-6736(03)14955-0

[bib11] Heimann G, Neuhaus G (1998) Permutational distribution of the log-rank statistic under random censorship with applications to carcinogenicity assays. Biometrics 54: 168–184 9544515

[bib12] Ho GY, Bierman R, Beardsley L, Chang CJ, Burk RD (1998) Natural history of cervicovaginal papillomavirus infection in young women. N Engl J Med 338: 423–428 9459645 10.1056/NEJM199802123380703

[bib13] Jacobs MV, Snijders PJ, van den Brule AJ, Helmerhorst TJ, Meijer CJ, Walboomers JM (1997) A general primer GP5+/GP6(+)-mediated PCR-enzyme immunoassay method for rapid detection of 14 high-risk and 6 low-risk human papillomavirus genotypes in cervical scrapings. J Clin Microbiol 35: 791–795 9041439 10.1128/jcm.35.3.791-795.1997 PMC229677

[bib14] Khan MJ, Castle PE, Lorincz AT, Wacholder S, Sherman M, Scott DR, Rush BB, Glass AG, Schiffman M (2005) The elevated 10-year risk of cervical precancer and cancer in women with human papillomavirus (HPV) type 16 or 18 and the possible utility of type-specific HPV testing in clinical practice. J Natl Cancer Inst 97: 1072–1079 16030305 10.1093/jnci/dji187

[bib15] Klein JP, Moeschberger ML (1997) Survival analysis: techniques for censored and truncated data. Sringer-Verlag: New York, USA

[bib16] Kotaniemi-Talonen L, Nieminen P, Anttila A, Hakama M (2005) Routine cervical screening with primary HPV testing and cytology triage protocol in a randomised setting. Br J Cancer 93: 862–867 16189520 10.1038/sj.bjc.6602799 PMC2361654

[bib17] Kulasingam SL, Hughes JP, Kiviat NB, Mao C, Weiss NS, Kuypers JM, Koutsky LA (2002) Evaluation of human papillomavirus testing in primary screening for cervical abnormalities: comparison of sensitivity, specificity, and frequency of referral. JAMA 288: 1749–1757 12365959 10.1001/jama.288.14.1749

[bib18] Lungu O, Sun XW, Felix J, Richart RM, Silverstein S, Wright Jr TC (1992) Relationship of human papillomavirus type to grade of cervical intraepithelial neoplasia. JAMA 267: 2493–2496 1349360

[bib19] Molano M, Van den BA, Plummer M, Weiderpass E, Posso H, Arslan A, Meijer CJ, Munoz N, Franceschi S (2003) Determinants of clearance of human papillomavirus infections in Colombian women with normal cytology: a population-based, 5-year follow-up study. Am J Epidemiol 158: 486–494 12936904 10.1093/aje/kwg171

[bib20] Peto J, Gilham C, Deacon J, Taylor C, Evans C, Binns W, Haywood M, Elanko N, Coleman D, Yule R, Desai M (2004) Cervical HPV infection and neoplasia in a large population-based prospective study: the Manchester cohort. Br J Cancer 91: 942–953 15292939 10.1038/sj.bjc.6602049 PMC2409880

[bib21] Richardson H, Kelsall G, Tellier P, Voyer H, Abrahamowicz M, Ferenczy A, Coutlee F, Franco EL (2003) The natural history of type-specific human papillomavirus infections in female university students. Cancer Epidemiol Biomarkers Prev 12: 485–490 12814991

[bib22] Sasagawa T, Basha W, Yamazaki H, Inoue M (2001) High-risk and multiple human papillomavirus infections associated with cervical abnormalities in Japanese women. Cancer Epidemiol Biomarkers Prev 10: 45–52 11205488

[bib23] van den Brule AJ, Pol R, Fransen-Daalmeijer N, Schouls LM, Meijer CJ, Snijders PJ (2002) GP5+/6+ PCR followed by reverse line blot analysis enables rapid and high-throughput identification of human papillomavirus genotypes. J Clin Microbiol 40: 779–787 11880393 10.1128/JCM.40.3.779-787.2002 PMC120256

[bib24] Walboomers JM, Jacobs MV, Manos MM, Bosch FX, Kummer JA, Shah KV, Snijders PJ, Peto J, Meijer CJ, Munoz N (1999) Human papillomavirus is a necessary cause of invasive cervical cancer worldwide. J Pathol 189: 12–19 10451482 10.1002/(SICI)1096-9896(199909)189:1<12::AID-PATH431>3.0.CO;2-F

